# Evidence of Formation of Superdense Nonmagnetic Cobalt

**DOI:** 10.1038/srep41856

**Published:** 2017-02-03

**Authors:** Nasrin Banu, Surendra Singh, B. Satpati, A. Roy, S. Basu, P. Chakraborty, Hema C. P. Movva, V. Lauter, B. N. Dev

**Affiliations:** 1 Department of Materials Science, Indian Association for the Cultivation of Science, 2A & 2B Raja S. C. Mullick Road, Jadavpur, Kolkata 700032, India; 2 Solid State Physics Division, Bhabha Atomic Research Centre, Mumbai 400085, India; 3 Surface Physics and Material Science Division, Saha Institute of Nuclear Physics, 1/AF, Bidhannagar, Kolkata 700064, India; 4 Microelectronic Research Center, The University of Texas at Austin, 10100 Burnet Road, Bldg 160, MER 1.606J, Austin, Texas 78758, USA; 5 Quantum Condensed Matter Division, Neutron Science Directorate, Oak Ridge National Laboratory, One Bethel Valley Road, Oak Ridge, TN 37831-6475, USA

## Abstract

Because of the presence of 3*d* transition metals in the Earth’s core, magnetism of these materials in their dense phases has been a topic of great interest. Theory predicts a dense face-centred-cubic phase of cobalt, which would be nonmagnetic. However, this dense nonmagnetic cobalt has not yet been observed. Recent investigations in thin film polycrystalline materials have shown the formation of compressive stress, which can increase the density of materials. We have discovered the existence of ultrathin superdense nonmagnetic cobalt layers in a polycrystalline cobalt thin film. The densities of these layers are about 1.2–1.4 times the normal density of Co. This has been revealed by X-ray reflectometry experiments, and corroborated by polarized neutron reflectometry (PNR) experiments. Transmission electron microscopy provides further evidence. The magnetic depth profile, obtained by PNR, shows that the superdense Co layers near the top of the film and at the film-substrate interface are nonmagnetic. The major part of the Co film has the usual density and magnetic moment. These results indicate the possibility of existence of nonmagnetic Co in the earth’s core under high pressure.

Interest in magnetism in 3*d* transition metals, iron, nickel and cobalt, has continued beyond the traditional bulk materials and thin films. Recent investigations have concentrated on two-dimensional monolayers, one-dimensional monatomic chains^1^ and even on single atoms on surfaces^2^. Even for the bulk material, due to the existence of magnetism at Earth’s core, the studies of structure and magnetism of 3*d* transition metals at reduced volume (under high pressure) have received a great deal of attention^3^. Theoretically, with decreasing atomic volume, the magnetic moment of Co decreases, and the magnetic moment of a fcc Co phase nearly vanishes when the atomic volume is reduced by a factor of ~1.4^4^,^5^. In a high pressure experiment on Co a hcp to fcc phase transition has been observed around this atomic volume corresponding to a density of ~1.4 times the normal Co density^3^. However, there was no magnetic measurement. No evidence of high density (HD) nonmagnetic Co has so far been reported. Polycrystalline thin films, on the other hand, can produce a compressive stress in the film^6^, thereby increasing the material density by mimicking high pressure conditions. Thus, polycrystalline thin films of Co offers a possibility to observe the HD nonmagnetic Co phase.

Here we report on the observation of a nonmagnetic fcc phase of cobalt, the density of which is about 1.2–1.4 times the density of normal hcp cobalt. We observe such a HD nonmagnetic phase in an electron-beam deposited polycrystalline Co thin (~25 nm) film on a Si(111) substrate. The evidence of a superdense cobalt phase is obtained from X-ray reflectivity (XRR) experiment. The result is corroborated by Rutherford backscattering spectrometry (RBS), secondary ion mass spectrometry (SIMS) and transmission electron microscopy (TEM) experiments. Additionally, the magnetic and the chemical depth profile analysis^7^,^8^,^9^ from polarized neutron reflectometry (PNR) experiments corroborate the high density and also show that this superdense cobalt layer is nonmagnetic.

## Results and Discussion

**Figure 1:**
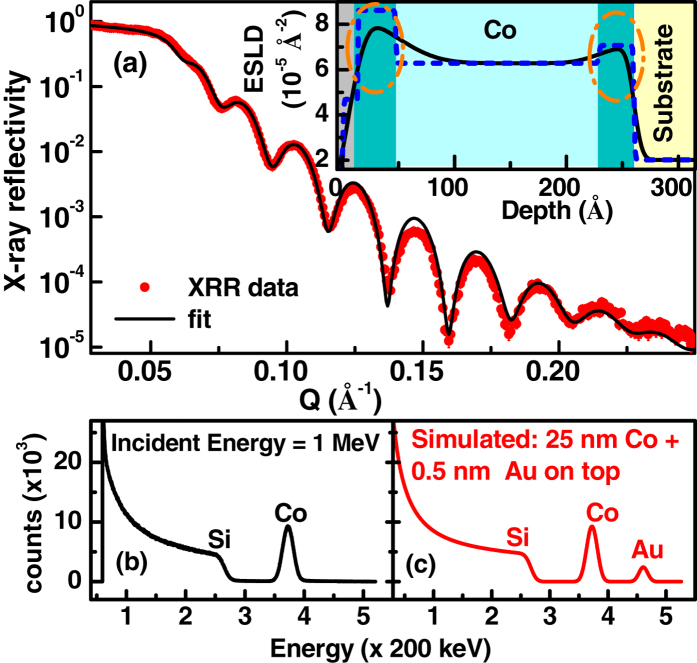
(**a)** X-ray reflectivity data (symbol) and fits considering the electron scattering length density (ESLD)model as shown in the inset. The ESLD depth profile (inset) with a uniform density Co layer and HD Co layers at the interfaces best fitted the XRR data. SLD histogram is shown as dashed line. HD Co regions are shown as deep green and also marked by ovals. (**b**) The RBS data from the sample. (**c**) The simulated RBS spectrum assuming a 0.5 nm Au layer on top of the Co layer.

XRR is a highly versatile and useful tool for the study of nano-structured thin films. It provides electron scattering length density (ESLD) or electron density profile and rms roughness parameters in the direction normal to the film plane^7^. Figure 1(a) shows XRR results. Insets show ESLD depth profiles used for fitting the XRR data. We obtained a total thickness of 270 ± 20 Å for the Co film from the XRR data. We could not fit the XRR data assuming a single uniform density Co layer along the whole depth of the film and thus considered different layer structures for fitting the XRR data. The details of different models and corresponding fits are shown in Fig. S1 of the Supplementary Information. The best fit (solid line, black) to the XRR data was achieved only by considering a ESLD profile that shows higher ESLDs at the Co/Si interface as well as near the Co/air interface, as shown in Fig. 1. ESLD, thickness and roughness parameters obtained from XRR are given in Table S1. We obtained an ELSD of (6.29 ± 0.10) × 10^−5^ Å^−2^ for the mid-depth-region of the Co film which is close to the ESLD of normal bulk Co (Table 1). The ESLD close to the Co/air interface of the Co film [~(8.56 ± 0.20) × 10^−5^ Å^−2^] is much higher (~1.35 times) than that of normal bulk Co. In order to identify the layer with higher ESLD at both the interfaces we have compared the extracted ESLD from XRR with the theoretical ESLDs (Table 1) of different possible compounds/alloys in the sample. Since the ESLD for cobalt oxides (CoO and Co_3_O_4_) are less than that of Co (Table 1), this rules out the possibility of the high ESLD layer as CoO or Co_3_O_4_. However a thin topmost layer (13 ± 2 Å), where a smaller ESLD is observed, is likely to be CoO (and/or Co_3_O_4_). [SIMS shows that it is CoO (see the Supplemental Information)] We also obtained a layer (34 ± 3 Å) with higher ESLD [(7.20 ± 0.15) × 10^−5^ Å^−2^] at the Co/Si interface. Again, comparing the ESLD of this layer with ESLD of possible Co-Si alloys (Table 1) we can conclude that this layer is also not a Co-Si alloy layer. The identity of the layer which is not an alloy or compound of Co, with an ESLD of ~8 × 10^−5^ Å^−2^ is an open question. The Co film investigated here is a polycrystalline film (shown latter in TEM). In a polycrystalline film, there is a possibility of formation of grains under compressive strain^6^ leading to a HD layer. It is a distinct possibility that the high ESLD layer is actually a HD Co layer with an average density of about 1.3 times the normal Co density. However, in order to confirm that this is indeed a HD Co layer, we need to rule out the presence of any HD element as an inadvertent contaminant in the sample. The presence of any such element was ruled out by RBS^10^ experiments.

The RBS result is shown in Fig. 1(b). Signals from Si and Co are seen. No other element of higher atomic mass (or higher Z) is present in the sample. Such elements would have produced counts at higher energies compared to Co. The RBS technique is sensitive to an equivalent of a fraction of one atomic layer for high Z elements (say Au) (see ref. 10). A simulated RBS spectrum for a small amount of Au (0.5 nm) on the Co/Si sample is shown in Fig. 1(c) suggesting high sensitivity to high-Z elements. The absence of any peak at higher energies than the Co peak in the RBS result rules out the presence of any elements of higher density than that of Co in the sample. So, the XRR and the RBS results confirm that the high ESLD layer is a HD Co layer. Further evidence of HD Co and negligible contaminants have been verified by SIMS measurements [see the Supplementary Material].

**Figure 2:**
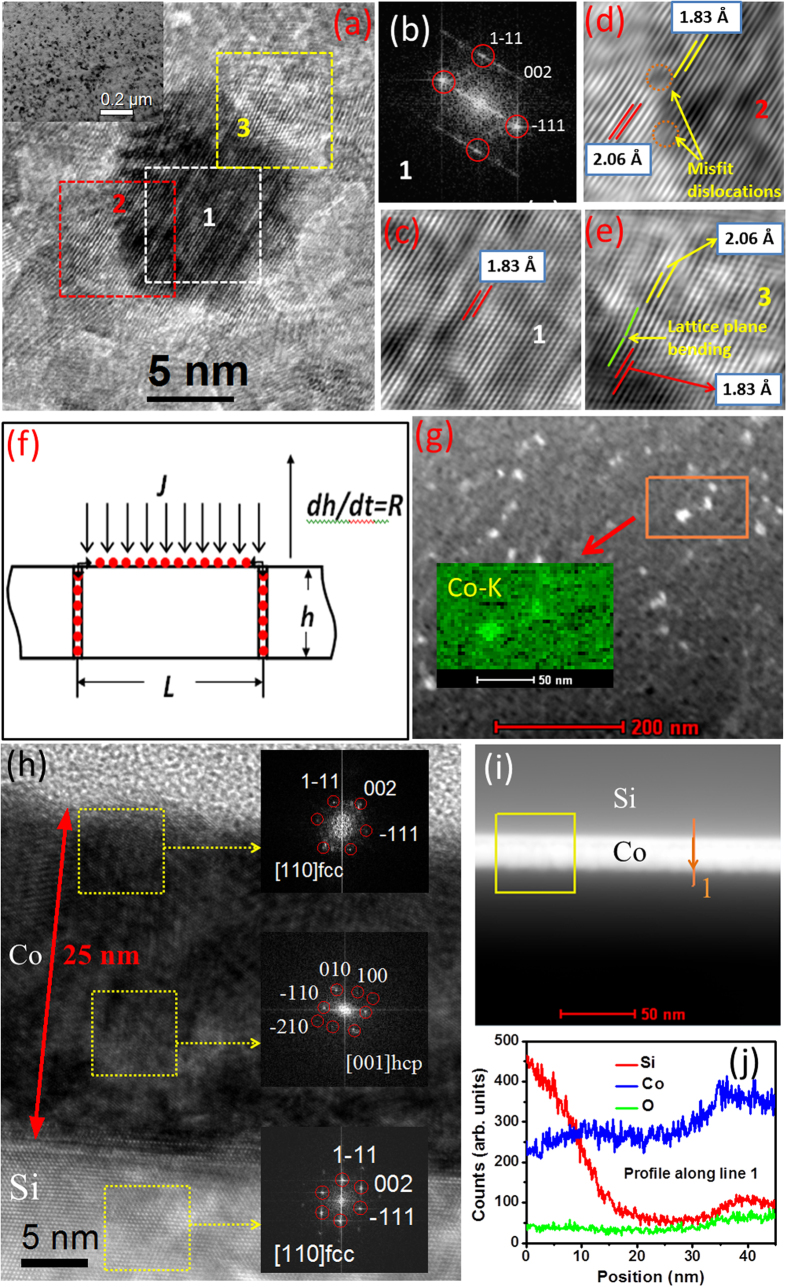
(**a**) Plan view high resolution TEM image, inset: a TEM image at lower resolution. (**b**) A FFT pattern from the boxed region ‘1’ in (**a**) reveals the fcc structure. (**c**) A Fourier-filtered lattice image from region ‘1’ in (**a**). (**d**) A lattice image from the boxed region ‘2’ in (**a**). (**e**) A lattice image from the boxed region ‘3’ in (**a**). (**f**) Schematic illustration of high density grain formation. (**g**) A STEM-HAADF image, inset: Co fluorescence map. (**h**) A XTEM image from the sample, insets: FFT patterns from different depth regions. (**i**) A cross-sectional STEM-HAADF image from the sample. (**j**) Drift-corrected X-ray fluorescence yield depth profile of Si, Co and O along the line marked in (**i**).

All experimental evidences, point to the formation of a superdense Co layer. We need to understand the mechanism of its formation. How does a HD phase form in a thin film, deposited under vacuum, and retain the higher density at ambient pressure? During the growth of a polycrystalline film via physical vapour deposition, deposited atoms can diffuse into grain boundaries producing a compressive stress on the grains^6^. This can be a possible reason for the formation of the superdense Co layer. This phenomenon is illustrated in Fig. 2(f), where *L* is the lateral size of a grain. The flux of deposited atoms is *J* and the growth rate is *dh*/*dt*. Deposited atoms can diffuse into grain boundaries, as shown, thereby producing a compressive stress in the grains, which would consequently have a higher density.

Obviously all the grains would not be under the same compressive stress, and consequently there would be a distribution of density. XRR determines the laterally averaged density for each depth. Plan view TEM image of our Co film [Fig. 2(a)] shows the polycrystalline grainy nature of the film. It also indicates a lateral density distribution. From Fig. 2(b), which is a fast Fourier transform (FFT) pattern from the region of the dark (dense) grain marked ‘1’ in (a), we also notice that such Co grains have a fcc structure. A Fourier-filtered lattice image from the boxed region ‘1’ in (a) is shown in Fig. 2(c). We notice the (111) planar spacing of 1.83 Å. Figure 2(d) shows a lattice image from the region ‘2’ in (a) representing the boundary region of the dark grain. Just outside the grain the (111) planar spacing is 2.06 Å and within the grain 1.83 Å. This represents about 11% contraction of the lattice parameter within the dark grain. We also notice two dislocations due to lattice mismatch across the grain boundary. Figure 2(e) shows a lattice image from region ‘3’ in (a). We again notice that across the grain boundary, the lattice parameters are 2.06 Å and 1.83 Å, outside and within the grain respectively. Lattice bending due to lattice mismatch is seen here. Figure 2(f) schematically shows the mechanism of formation of compressed (dense) grains, as explained in the previous paragraph. Figure 2(g) shows a scanning transmission electron microscopy high-angle annular dark field (STEM-HAADF) image. We notice lateral variation of Co density and the presence of some very high density Co grains (bright). Energy dispersive x-ray (EDX) map of Co (inset) also shows intense Co fluorescence from the bright grains, reflecting their higher density, compared with their surroundings, which are also mainly HD Co. The cross-sectional TEM (XTEM) image in Fig. 2(h) along with FFTs from different depth regions of the sample shows that the superdense top layer of the Co film is fcc. This is consistent with theory^4^. The mid-depth region of the Co film of normal density shows hcp structure. The substrate Si is fcc, as expected. Figure 2(i) shows a cross-sectional STEM-HAADF image of the sample. In HAADF image a high-Z material looks brighter. That is why the Co layer looks brighter than Si. Depth profiles of X-ray fluorescence yields of Si, Co and O along the marked line in (i) are shown in Fig. 2(j). The Co fluorescence depth profile indicates the presence of HD Co near the Co/air interface as well as some density enhancement near the Co/Si interface, consistent with the XRR results.

**Figure 3:**
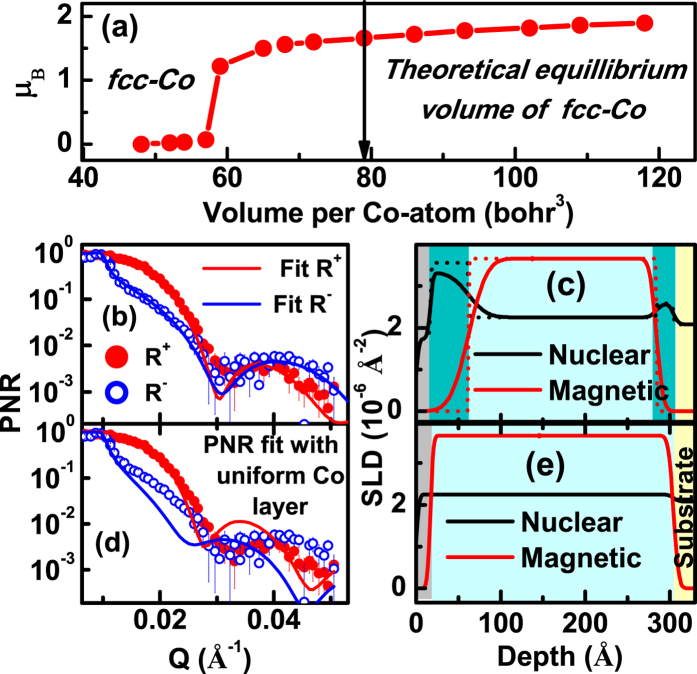
(**a**) Magnetic moment (μ_**B**_) as a function of atomic volume of fcc Co (adapted from ref. 4). (**b**) PNR data (symbols) and fits (solid lines) considering nuclear and magnetic scattering length density (NSLD and MSDL) models shown in (**c**),which corresponds to HD Co layers at interfaces. SLD histograms are also shown in (c) as dashed lines. HD Co regions are shown as deep green. (**d**) PNR data and fits considering the NSLD and MSLD models, corresponding to uniform density of Co, shown in (**e**).

The variation of magnetic moment as a function of volume, as suggested by theoretical prediction^4^ is reproduced in Fig. 3(a). As we have observed a superdense fcc phase of Co, we have also investigated its magnetic properties. Macroscopic magnetic measurements would provide the magnetic contribution from the entire Co film and would not be able to distinguish the magnetic contribution from these superdense ultrathin Co layers. So we carried out magnetic depth profiling by PNR experiments^7^,^8^,^9^, on the same sample as the one used in XRR experiment. PNR experiments were carried out using the neutron reflectometer in DHRUVA, Bhabha Atomic Research Centre, Mumbai, India^11^. PNR data (symbols) and fit (solid lines) are shown in Fig. 3(b) and (d) for the nuclear SLD (NSLD) and magnetic SLD (MSLD) profile models shown in Fig. 3(c) and (e), respectively. A uniform NSLD and MSLD (uniform density Co layer) depth profile as shown in Fig 3 (e) does not fit the PNR data (Fig. 3(d)) over the entire Q range, suggesting that the uniform density depth profile of Co is unacceptable. Best fit to PNR data (Fig. 3 (b)) is obtained from the NSLD/MSLD depth profile (Fig. 3(c)) which is consistent with XRR results. The NSLD [(2.26 ± 0.07) × 10^−6^ Å^−2^] obtained from PNR for the mid-region of the depth profiles [Fig. 3 (c)] is consistent with normal Co (Table 1). We observed higher NSLDs of (3.30 ± 0.15) × 10^−6^ Å^−2^ and (2.70 ± 0.14) × 10^−6^ Å^−2^ for the interface layers at air/Co and Co/Si interfaces, respectively. Comparing these NSLDs with the NSLDs of possible compounds/alloys at these interfaces (Table 1) as well as the results obtained from XRR, RBS and SIMS analysis (see Supplemental Information) it is confirmed that these layers at the interfaces are HD Co layers. However the top layer at air/film interface with thickness of 10 ± 2 Å show reduced NSLD [(1.7 ± 0.12) × 10^−6^ Å^−2^], which may be due to an oxide layer, and the reduction in this NSLD as compared to the NSLD of Co oxide might have resulted due to higher roughness (~10 Å) at the surface. PNR additionally shows that the HD Co layer at both interfaces has nearly zero MSLD or magnetic moment density. The normal Co layer shows a marginally lower value of magnetization ~1250 kA/m [MSLD (3.65 ± 0.12) × 10^−6^ Å^−2^] as compared to its bulk value (~1400 kA/m).

**Figure 4:**
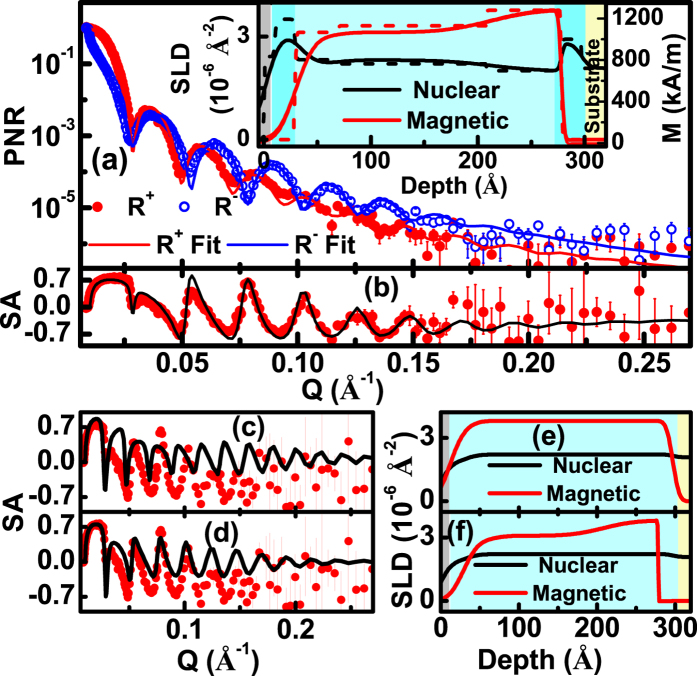
(**a**,**b**) Show the PNR and SA data (symbols) with best fits (solid lines) corresponding to NSLD and MSLD depth profiles shown in the inset. SLD histograms are also shown in the inset as dashed lines. (**c**,**d**) Represent the SA data and fit corresponding to SLD models with NSLD intentionally fixed to a uniform value as shown in (**e**,**f**), respectively. In both (**c**,**d**) the fit considerably deviates from the data.

For an additional confirmation of these PNR results, we performed independent measurements at the Magnetism Reflectometer^12^ at the SNS, ORNL USA, in a broad Q-range to obtain a higher spatial resolution. The sample for PNR experiment at ORNL is cut from a different part of the Co film grown on a Si wafer, although from the same wafer. These results are shown in Fig. 4. The NSLD and the MSLD [inset of Fig. 4(a)], have similar nature to those in Fig. 3 suggesting higher NSLD with no ordered ferromagnetism in Co near the air/Co and the Co/Si interfaces. There are some differences in the details of the NSLD and the MSLD depth profiles compared to the results obtained from the measurement at DHRUVA. The reasons for the differences are twofold. Firstly, these results are not from the same part of the sample. Another reason is, due to the higher Q range covered at SNS we have better spatial resolution for a refinement of the parameters for fitting the PNR data. In spite of the differences in the Q-ranges, both the experiments at DHRUVA and at ORNL endorse the existence of HD Co layers at the air/film and the film/Si substrate interfaces. The fit of ORNL data suggests some variation in magnetization in the bulk Co layer. As we see from the plan-view TEM images (Fig. 2a), the density of the Co layer is not laterally uniform. Therefore, measurements carried out on samples, cut from different regions of the wafer, may yield some variation in results. From the SLD profiles in the inset of Fig. 4(a) we notice a depth region of the Co film close to the Co/Si interface, where the density of Co (NSLD) is somewhat lower than that of normal Co with marginally higher MSLD. This is consistent with the theoretical result, as seen in Fig. 3(a) that lower density will cause an increase of magnetic moment in Co. Fig. 4(b) shows the spin asymmetry (SA) data [defined as (R^+^ − R^−^)/(R^(+)^ + R^(−)^), where R^±^ are spin dependent reflectivity] and the corresponding fit for the SLDs in Fig. 4(a). Figure 4(c) and (d) are SA data and fits assuming two different models: (i) uniform NSLD and MSLD depth profiles as shown in (e) and (ii) uniform NSLD and non-uniform MSLD profiles as shown in (f), respectively. It is evident from Fig. 4(c–f) that these models do not fit the SA data. Thus all the three reflectometry results, XRR, PNR at DHRUVA and at ORNL clearly confirm the existence of a higher density Co layer at air/Co and Co/substrate interface with nearly zero magnetic moment density.

We have also investigated Co films with a protective Au layer (2 nm) on top in order to avoid surface oxidation of Co. We have studied several film thicknesses of Co, such as 25 nm, 22 nm, 12 nm and 4 nm. In all cases we have observed HD Co at the interfaces.

We conjecture the following to understand the enhanced density of Co that has been observed near the top surface and at the Co/Si interface of the Co film. At the beginning of Co deposition on Si, due to higher surface free energy of Co compared to Si, Co would tend to grow as islands. When islands coalesce and further deposited atoms enter into grain boundaries, they would generate a compressive stress on the Co grains, thereby enhancing the material density^6^. Thereafter, the growth is Co on already deposited Co layer. The polycrystalline Co growth would continue. If the grain density increases near the top and more atoms enter into these grain boundaries that would increase the density near the top of the film. However, to understand the mechanism of the formation of a superdense phase of material in thin films, detailed investigations depending on growth conditions, such as deposition rate, growth temperature etc., are necessary. Similar enhancement effect, both at the interface and near the top of a thin film, has been observed in other systems, which could be understood in terms of surface free energy differences^13^.

When the results are compared to the bulk Co, several differences are noticed. At room temperature and ambient pressure bulk Co has hcp structure. Fcc structure of bulk Co has been observed under high pressure corresponding to densities in the range of 1.3–1.5 times the normal density^3^. (No magnetic measurements were reported in ref. 3). In the present case, for the thin Co film no external pressure was applied; yet high density fcc phase is formed. The pressure in thin films is internal due to compressive stress. Also density functional theory (DFT) calculation for bulk Co predicts the loss of magnetism of fcc Co at a density of about 1.4 times the normal density^4^. In the Co thin film we observe loss of magnetism in the high density Co for densities ~1.2–1.4 times the normal density. Usually, materials in the form of thin films may show structures and properties quite different from their respective bulk form^14^. Under ambient conditions, thin films may have structures which either do not exist in nature or exist only under extreme conditions of temperature and/or pressure for the corresponding bulk material^15^. For bulk cobalt, fcc structure exists at temperatures above 417 °C^16^ or under high pressure^3^. However, in thin films cobalt may exist in fcc structure at room temperature and normal pressure and their properties vary widely compared to bulk Co^14^. A mixed hcp and fcc phase exists in bulk cobalt only under high pressure, over a large pressure range^3^. However, in thin films a mixed hcp and fcc phase exists at room temperature and normal pressure^17^. Therefore the pressure or temperature dependence of the structures and properties of a bulk material cannot be directly compared with those of thin films. In some cases the differences between thin film and bulk are very drastic. For example, Bi is not superconducting at ambient pressure; it is superconducting under high pressure (Tc about 8 K)^18^. However, thin films of Bi is superconducting at ambient pressure (Tc about 5–6 K)^19^. Thin films provide extra degrees of freedom which may drastically change the environmental variables required to get phase transitions in thin films. Thus, the formation of high density Co in thin films without the application of external pressure, though different from the behavior of bulk Co, is not surprising.

Loss of ferromagnetism in dense Co has been predicted by DFT calculation^4^. DFT calculations have also predicted loss of ferromagnetism for other magnetic 3d transition metals^20^,^21^. The mechanism for loss of magnetism as a function of pressure (or density) is explained in details by Zeng *et al*. from their DFT calculations^20^. The unpaired 3d electrons are forced to be paired under pressure thereby reducing the magnetic moment. Their work shows the pressure dependence of the electronic density of states (DOS) for up-spin and down-spin. The asymmetry between up-spin and down-spin DOS (reason for magnetic moment) gradually becomes nearly symmetric (reason for nearly zero magnetic moment) with increasing pressure.

## Conclusion

Depth profiles of electron scattering length density (ESLD) obtained from XRR, and nuclear scattering length density (NSLD) from PNR, in a polycrystalline cobalt thin film on silicon have revealed regions of cobalt with much higher density compared to normal cobalt. These regions are near the air/Co and the Co/Si interfaces. The density of the dense layer near the air-film interface is about 1.3–1.4 times that of normal Co and its structure is fcc and not the hcp structure seen in bulk Co. Below this layer the film is hcp Co of usual density. As revealed by the depth profile of the magnetic scattering length density (MSLD), the superdense Co layer is nonmagnetic. Usually such a high density phase can only be formed under high pressure. However, during the growth of a polycrystalline film via atomic deposition, a compressive residual stress on polycrystalline grains due to incorporation of atoms in the grain boundaries can possibly be the mechanism to form high density material. Apparently, that is how this high density layer has formed. The evidence for nonmagnetic superdense Co indicates the possibility of existence of nonmagnetic Co in the earth’s core under high pressure.

## Experimental Methods

A thin cobalt film (25 nm) was deposited on piranha cleaned, HF-etched Si(111) substrate in high vacuum by electron-beam evaporation method. Then the cobalt film is taken out of the vacuum chamber. The exposure of the film to air led to some surface oxidation. X-ray reflectometry (XRR) experiment was carried out with Cu K_α_ X-rays. We have carried out polarized neutron reflectivity (PNR) experiment on this cobalt film using the neutron reflectometer in DHRUVA, Bhabha Atomic Research Centre, Mumbai, India, which uses neutrons of wavelength 2.5 Å^11^. Because of low neutron intensity at the DHRUVA facility, the accessible momentum transfer (Q) range is quite small. PNR experiment was repeated at the Oak Ridge National Laboratory facility where the accessible momentum transfer range is large. Rutherford backscattering spectrometry (RBS), secondary ion mass spectrometry (SIMS) and cross sectional transmission electron microscopy (XTEM) experiments were carried out in order to remove any ambiguity in the interpretation of the XRR and PNR data.

XRR and PNR are non-destructive techniques from which the depth dependent structure of the sample with sub-nanometer resolution averaged over the lateral dimensions of the entire sample (typically 100 mm^2^) can be inferred^7^,^8^,^9^. XRR and PNR involve measurement of the x-ray/neutron radiation reflected from a sample as a function of wave vector transfer *Q* (i.e., the difference between the outgoing and incoming wave vectors). In case of specular reflectivity (angle of incidence = angle of reflection) 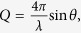 where θ is the angle of incidence and λ is the wavelength of x-ray/neutron, and it is qualitatively related to the square of the Fourier transform of the scattering length density (SLD) depth profile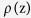 (normal to the film surface or along the z-direction)^7^,^8^,^9^. For XRR, 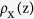 is proportional to electron density whereas for PNR, 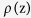 consists of nuclear and magnetic SLDs such that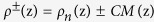, where *C* = 2.9109 × 10^−9^ Å^−2^ m/kA, and *M*(z) is the magnetization (kA/m) depth profile^7^,^8^,^9^. The sign +(−) is determined by the condition when the neutron beam polarization is parallel (opposite) to the applied field and corresponds to reflectivities *R*^±^.

RBS experiment was carried out with 1 MeV He^+^ ions. Scattered ions were detected at a scattering angle of 165 degree. RBS can detect the presence of any high-Z element with very high sensitivity (about a hundreth of an atomic layer). RBS is a non-destructive technique.

SIMS experiments were carried out by sputtering the sample with 3 keV Ar^+^ ions at an ion beam current of 15 nA and detecting various species of sputtered molecular ions. Low beam current density (beam current/rastered area) was chosen to ensure low sputter-erosion rate so as to improve the SIMS depth resolution. As a function of time the sputtered ions come from different depths thereby providing the depth profile.

TEM measurements were carried out with a 300 keV electron beam. Plan view TEM, XTEM, STEM-HAADF imaging as well as X-ray imaging were carried out. Elemental depth profile was obtained via Co, Si and O fluorescence measurement from different depths.

## Additional Information

**How to cite this article**: Banu, N. *et al*. Evidence of Formation of Superdense Nonmagnetic Cobalt. *Sci. Rep.* **7**, 41856; doi: 10.1038/srep41856 (2017).

**Publisher's note:** Springer Nature remains neutral with regard to jurisdictional claims in published maps and institutional affiliations.

## Supplementary Material

Supplementary Information

## Figures and Tables

**Table 1:** Bulk (theoretical)^8^ value of scattering length density (SLD) of the Co, cobalt oxides and cobalt silicides for X-rays (ρ_x_, ESLD) and neutron (ρ_n_, NSLD).

Composition	Co	CoO	Co_3_O_4_	CoSi	CoSi_2_
ρ_x_ (10^−5^ Å^−2^)	6.30	4.76	4.56	5.00	4.1
ρ_n_ (10^−6^ Å^−2^)	2.27	4.29	4.69	3.01	2.99
