# Supplementary material for: Evidence of Formation of Superdense Nonmagnetic Cobalt
Source: Sci Rep. 2017 Feb 3;7:41856. doi: 10.1038/srep41856 (PMC5291096; doi:10.1038/srep41856)
Supplement: Supplementary Information [file srep41856-s1.pdf]

Supplementary Material

Evidence of Formation of Superdense Nonmagnetic Cobalt

Nasrin Banu[1], Surendra Singh[2], B. Satpati[3], A. Roy[4], S. Basu[2], P. Chakraborty[3], Hema C. P. Movva[4], V. Lauter[5] and B. N. Dev[1*]

A thin cobalt film (25 nm nominal thickness) was deposited on piranha cleaned, HF-etched Si(111) substrate (a 100 mm dia wafer) in high vacuum by electron-beam evaporation method. Then the cobalt film was taken out of the vacuum chamber. The exposure of the film to air led to some surface oxidation. X-ray reflectivity (XRR) experiment was carried out with Cu K_α X-rays. We have carried out polarized neutron reflectivity (PNR) experiment on this cobalt film using the neutron reflectometer in DHRUVA, Bhabha Atomic Research Centre, Mumbai, India, which uses neutrons of wavelength 2.5 Å [1]. PNR experiment was repeated with the Magnetism Reflectometer at the spallation neutron source (SNS), Oak Ridge National Laboratory [2], where the accessible Q range is large. Rutherford backscattering spectrometry (RBS), secondary ion mass spectrometry (SIMS) and transmission electron microscopy (TEM) including cross-sectional TEM experiments were carried out in order to remove any ambiguity in the interpretation of the PNR and XRR data.PNR and XRR data showed existence of high density non-magnetic Co layers at the air-film and film-substrate interfaces. Using RBS, SIMS and TEM we have showed that the high density phase is an fcc phase of Co.

X-ray and polarized neutron reflectivity:

XRR and PNR are non-destructive techniques from which the depth dependent structure of the sample with sub-nanometer resolution averaged over the lateral dimensions of the entire sample (typically 100 mm^2) can be obtained [3-5]. XRR and PNR involve measurement of the x-ray/neutron radiation reflected from a sample surface as a function of wave vector transfer Q (i.e., the difference between the outgoing and incoming momentum vectors). In case of specular (angle of incidence = angle of reflection) reflectivity (R) $Q = \frac{4\pi}{\lambda} sin\theta,$ where θ is the angle of incidence and λ is the wavelength of x-ray/neutron. R(Q) is quantitatively related to the square of the Fourier transform of the scattering length density's (SLD) depth profile $\rho(\mathrm{z})$ (normal to the film surface or along the z-direction) [3-5]. For XRR, $\rho_{\mathrm{x}}(\mathrm{z})$ is the electron SLD (ESLD), which is proportional to the electron density profile whereas for PNR, $\rho(\mathrm{z})$ consists of nuclear and magnetic SLDs (NSLD and MSLD) such that $\rho^{\pm}(\mathrm{z}) = \rho_n(\mathrm{z}) \pm CM(\mathrm{z})$, where C = 2.9109×10^{-9} $Å^{-2}$ m/kA, and M(z) is the magnetization (kA/m) depth profile [3-5]. The sign +(-) is determined by the condition when the neutron beam polarization is parallel (opposite) to an applied magnetic field and corresponds to reflectivities, $R^{\pm}$. ESLD and NSLD are proportional to material density. So the ESLD and NSLD depth profiles reflect the density depth profile.

The chemical density depth profiles were obtained from XRR data by fitting a model $\rho(\mathrm{z})$ whose reflectivity best fit the data. The reflectivities were calculated using the dynamical formalism of Parratt [6], and parameters of the model were adjusted to minimize the value of reduced χ^2—a weighted measure of goodness of fit [7]. To fit XRR data, we considered different model structures consisting of layer(s) representing regions with different ESLD [3-5]. The parameters of the model also included layer thickness and interface (or surface) roughness [3-5].

Fig. S1 shows XRR data and corresponding fits for three ESLD models. Fig. S1 (a) , (b) and (c) show the XRR data (solid circles) and corresponding fits (solid black lines) for ESLD models shown in Fig. S1 (d), (e) and (f), respectively. Fig. S1 (d), (e) and (f) show ESLD models which corresponds to a sample structure of (1) A low density oxide layer at air-film interface and a uniform desnity Co layer below it which can be

represented as: CoO/Co/Si (substrate), (2) CoO/Co/Co(high density, HD)/Si (substrate) and (3) CoO/Co(HD)/Co/Co(HD)/Si (substrate), respectively. The minimum reduced χ^2 values for models (1), (2), and (3) were 27, 16, and 3.5, respectively. Of the three models, model (3) (four layer model, in Fig. S1(f)) produced best fit to the data. The best fit to XRR data clearly suggests formation of high density (HD) Co layer at both CoO/Co and Co/Si interfaces. The parameters (Fig. S1 (f)) obtained from the best fit (Fig. S1(c)) of XRR data are given in Table S1. The dashed blue lines in Fig. S1 (d –f) are ESLD profiles which are modulated with interface roughness as an error function [4].

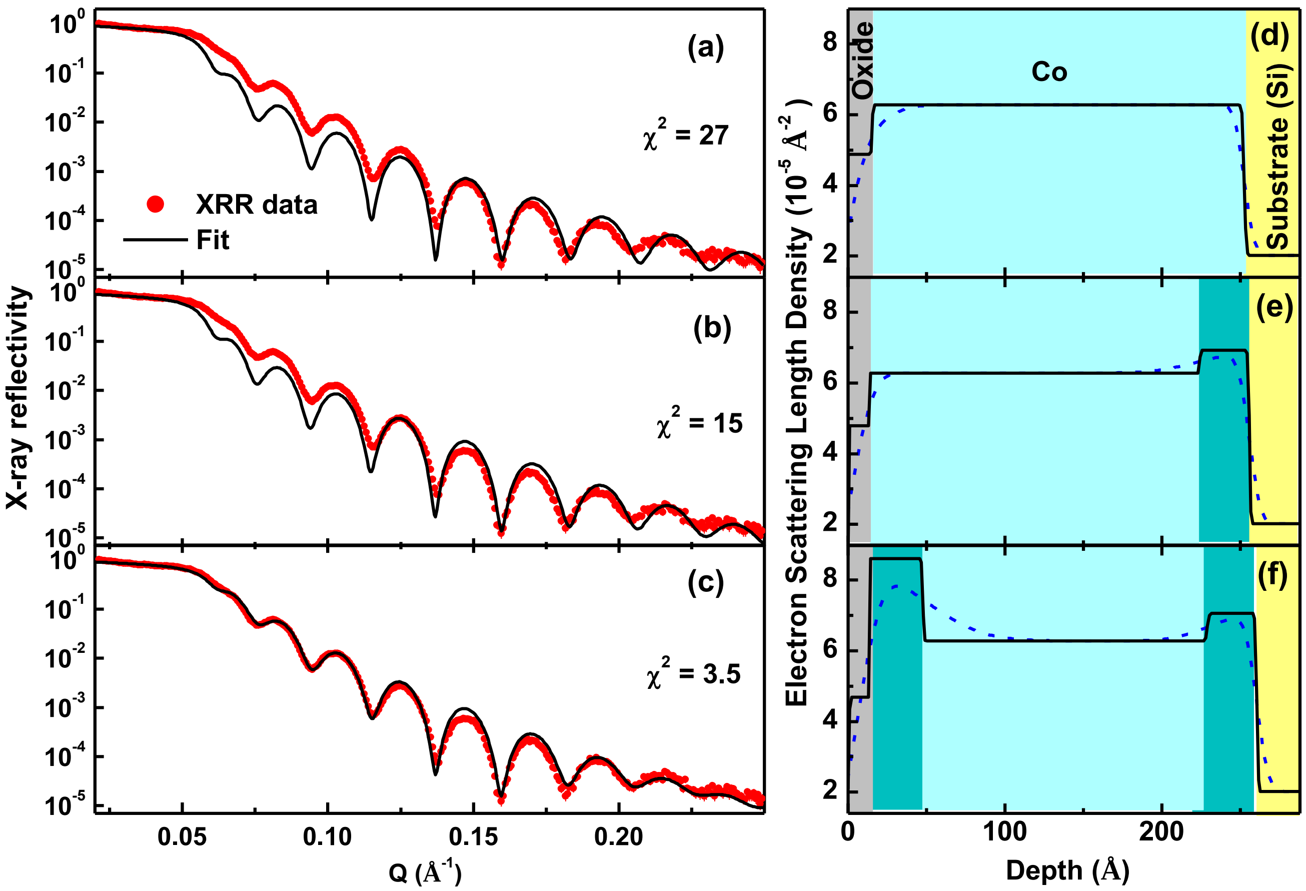

Fig. S1: X-ray reflectivity data (symbols) and fits considering three electron scattering length density (ESLD) models. The three ESLD models are, (1) a uniform density Co layer, (2) uniform density Co layer and high density (HD) Co layer at Co/Substrate interface and (3) a uniform density Co layer with HD Co layers at both Air/Co and Co/substrate interfaces, shown in (d), (e) and (f), respectively and corresponding fits to XRR data are shown in (a), (b) and (c), respectively.

Table S1: Depth dependent parameters obtained from XRR measurements from Co film.

layers	Thickness (Å)	ESLD (10^{-5} Å^{-2})	Roughness (Å)
CoO	13±2	4.68±0.10	7±2
HD Co	35±3	8.56±0.20	8±2
Co	183±5	6.28±0.10	24±4
HD Co	34±3	7.20±0.15	13±3
Si substrate	-	2.05±0.05	5±2

From XRR measurements we obtained HD Co of thickness (~ 35 Å) at both Co/Si and near air/Co interface which show different ESLD of $(7.20\pm0.15)\times10^{-5}$ Å^{-2} and $(8.56\pm0.20)\times10^{-5}$ Å^{-2}, respectively, which are much higher than the ESLD of hcp Co and possible phases (Co oxide and Co silicides). Thus XRR clearly suggests formation of HD Co at interfaces.

RBS experiment was carried out with 1 MeV He^+ ions. Scattered ions were detected at a scattering angle of 165 degree. RBS [8] can detect the presence of any high atomic number (high-Z) element with very high sensitivity (about 10^{13} atoms/cm^2, or equivalent to about a hundredth of an atomic layer). RBS is a nondestructive technique. In order to rule out the possibility of contamination by any high-Z (high density) material, we carried out RBS experiment. The RBS result has been discussed in the main paper. Signals from Si and Co are seen in the spectrum. No other element of higher atomic mass (or higher Z) is present in the sample. If they were present they would appear as peak(s) at higher energies than that of the position of the Co peak. It should be mentioned here that under the experimental condition the RBS technique is sensitive to an equivalent of a fraction of one atomic layer for high-Z elements (say Au). A simulated RBS spectrum for a small amount of Au (0.5 nm) on the Co/Si sample, for the same experimental condition, is also shown in the main paper in order to give an indication of the sensitivity of RBS to high-Z elements. We have taken 0.5 nm Au (~ $2x10^{15}$ atoms/cm^2) in the simulation so that the Au signal is prominent in the same scale as the Co signal. However, even ~ 10^{13} atoms/cm^2 of Au would produce a detectable signal. In the simulation we have taken Au on top of the Co layer. However, if this Au layer is anywhere within the Co film or distributed uniformly, it would produce the same signal. Absence of any high-Z element in the RBS spectrum confirms that the high ESLD layer observed in XRR is actually a high density Co layer.

SIMS results

SIMS experiments [9] were carried out by sputtering the sample with 3 keV Ar+ ions at an ion beam current of 15 nA and detecting various species of sputtered molecular ions. Low beam current density (beam current/rastered area) was chosen to ensure low sputter-erosion rate so as to improve the SIMS depth resolution. As a function of time the sputtered ions come from different depths thereby providing the depth profile.

We carried out SIMS experiments and detected different sputtered atomic and molecular ions from the sample. Again SIMS data did not show any evidence for the presence of any high-Z elements in the sample. SIMS data are shown in Fig. S2(a-b). In SIMS depth profile, secondary ion-yield of an individual species from the surface and interfaces are seen to get enhanced due to the presence of any impurity element in the matrix (the so-called matrix effect). Data from any depth can be safely interpreted provided the matrix effect is properly compensated. In Fig. S2(a-b) the time axis is proportional to depth. In Fig. S2(a) we notice the flat region of the Co yield which comes from the mid-region of the Co film. Ignoring the initial peak at the top surface, arising due to a ‘matrix effect’ in conventional SIMS, we notice that there is an enhanced Co yield in the outer region of the Co film, which is also a probable indication of a high density Co. This feature is more clear from Fig. S2(b), which shows the yield in an expanded time scale up to about $1.5x10^3$ sec. SIMS data show that there is a very thin layer of CoO at the top of the film and other oxides of Co are practically absent. A negligible amount of CoSi was detected by SIMS at the Co/Si interface [Fig. S2(a)]. We have to keep in mind that SIMS is the most sensitive surface analysis technique. While most of the techniques would not be able to detect below one (1) atomic layer (monolayer) of material, SIMS can detect an equivalent of ~10^{-9} monolayer material.

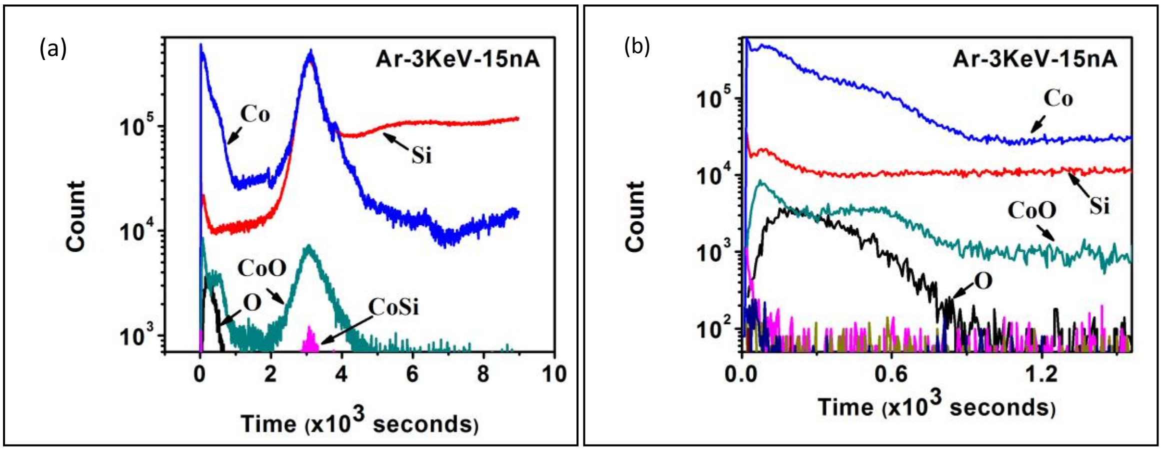

Fig. S2. (a), (b) SIMS depth profile of yield of different sputtered species (Co, Si, CoO, O; yields of CoSi, Co_2Si, Co_2O_3, Co_3O_4 are very low). Sputtering time is proportional to the depth in the sample. (c) Depth profile closer to the surface; for time larger than ~1000 sec, yield from normal Co is seen. For time less than 1000 sec, higher yield of Co is indicative of high density Co near the surface. (Note the logarithmic scale of yield of various species).

Other measurements

PNR measurements were also carried out at ORNL under other conditions. Measurements at 70 K (low temperature PNR) did not show any change of results. Field annealing at 473 K for 30 minutes with 1T magnetic field, followed by PNR measurement at room temperature did not reveal any significant change as well. In order to avoid the possibility of cobalt silicide formation, we did not choose a higher annealing temperature. These show that the high density Co phase is reasonably stable.

1. S. Basu and Surendra Singh, J. Neutron Res.**14**, 109 (2006).
2. V. Lauter, H. Ambaye, R. Goyette, W.-T. Hal Lee and A. Parizzi, Physica B, **404**, 2543 (2009).
3. H. Zabel, Appl. Phys. A, **58**, 159 (1994).
4. M. R. Fitzsimmons and C. Majkrzak, Modern Techniques for Characterizing Magnetic Materials, (Springer, New York, 2005), Chap. 3, pp.107–155.
5. Surendra. Singh, S. Basu, M. Gupta, C. F. Majkrzak and P. A. Kienzle, Phys. Rev. B **81,** 235413 (2010).
6. L. G. Parratt, Phys. Rev. 95, 359 (1954).
7. W. H. Press, B. P. Flannery, S. A. Teukolsky, and W. T. Vetterling, Numerical Recipes in Fortran: The Art of Scientific Computation, 2nd ed. (Cambridge University Press, Cambridge, 1992). 222205-4 Singh et al. J. Appl. Phys. 116, 222205 (2014).
8. W.K. Chu, J. W. Mayer, and M. A. Nicolet, Backscattering Spectrometry (Academic Press, 1978) pp. 124-126.
9. A. Benninghoven, F. G. Rudenauer, and H. W. Werner, Secondary Ion Mass Spectrometry: Basic Concepts, Instrumental Aspects, Applications, and Trends (Wiley, New York, 1987).
